# Activity-related behavior typologies in youth: a systematic review

**DOI:** 10.1186/s12966-019-0804-7

**Published:** 2019-05-16

**Authors:** Kate E. Parker, Jo Salmon, Sarah A. Costigan, Karen Villanueva, Helen L. Brown, Anna Timperio

**Affiliations:** 1 Institute for Physical Activity and Nutrition (IPAN), School of Exercise and Nutrition Sciences, Deakin University, Geelong, Australia; 2 School of Exercise and Nutrition Sciences, Deakin University, Geelong, Australia; 3 Centre for Urban Research, School of Global Urban and Social Studies, RMIT University, Melbourne, Australia

**Keywords:** Typologies, Physical activity, Sedentary behavior, Adolescents, Correlates

## Abstract

**Background:**

Clusters of adolescents differentiated by patterns of physical activity and sedentary behavior (activity-related typologies) are common. Understanding both the characteristics of adolescents and modifiable correlates of these typologies, can help to develop interventions for those most at risk. This systematic review aimed to synthesize the socio-demographic characteristics and modifiable correlates of activity-related behavioral typologies among adolescents.

**Methods:**

A systematic search of seven electronic databases was conducted to identify quantitative studies using person-oriented statistical approaches to identify activity-related behavioral typologies among 12–18 year-olds. This systematic review was registered in Prospero (registration number: CRD42016046879).

**Results:**

Thirty-six studies met the inclusion criteria and were classified according to three sub-themes based on behaviors included in the typologies (1. physical activity and sedentary behavior only; 2. physical activity, sedentary behavior and risk-related behaviors; 3. physical activity, sedentary behavior and diet). Studies were mostly cross-sectional and relied on self-report measures. Methods were considerably heterogeneous, however results revealed some consistency in typologies within specific groups. For example, typologies characterized by unhealthy behavior patterns (e.g., characterized by physical inactivity, high sedentary behavior and poor diet or high risk-related behaviors) comprised more older adolescents. With the exception of socio-demographics (age, sex, body mass index and socio-economic status), very few correlates have been studied to date (mostly school-related behavioral factors and intrapersonal influences), with evidence largely from typologies comprised of physical activity, sedentary behavior and diet.

**Conclusions:**

More research is needed to assess a range of modifiable correlates associated with activity-related behavior typologies among adolescents. This will allow for more targeted interventions, to achieve long-lasting, positive behavior change in adolescent populations.

**Electronic supplementary material:**

The online version of this article (10.1186/s12966-019-0804-7) contains supplementary material, which is available to authorized users.

## Background

Non-communicable diseases including obesity, type two diabetes and cardio-metabolic syndrome, are increasing among adolescent populations, particularly in Western countries [1]. Inadequate amounts of leisure-time physical activity and active travel, and high levels of sedentary behaviors (any waking behavior characterized by an energy expenditure ≤1.5 metabolic equivalents, while in a sitting, reclining or lying posture [2]) contribute to the risk of developing such diseases [1, 3]. In Australia and other developed countries, national statistics suggest that as many as 80% of adolescents do not achieve the physical activity guidelines of 60 min per day of moderate-to-vigorous physical activity [4]. Similarly, 80% of adolescents exceed the screen-time guidelines of no more than 2 h per day [4]. Such statistics are concerning given evidence suggests that these health compromising behaviors tend to track through to adulthood [5, 6].

Research has shown that adolescents do not necessarily displace time spent in physical activity with sedentary behaviors or vice versa, and looking at these behaviors in isolation may not allow for a complete profile of their activity-related behavior engagement [7, 8]. For example, those who engage in high levels of sport may also watch excessive amounts of television. With this in mind, researchers have begun to identify unique groups or ‘typologies’ of youth based on engagement in combinations of activity-related behaviors across the full activity spectrum [9]. The data-driven techniques distinguish groups of people who share similar patterns of behavior or characteristics [10]. Identifying typologies is attractive as it allows complex patterns of behavior to be characterized [11], better targeting of ‘audiences’ for behavior change interventions once the socio-demographic characteristics of individuals within typologies are established [12] and formulation of strategies that may prevent or promote a particular pattern of behavior [11] once correlates of typologies are established. Although research on typologies of activity-related behaviors among young people is limited, emerging evidence shows that these behaviors may have synergistic effects on health outcomes [13, 14], highlighting the need to target multiple behaviors simultaneously to gain the greatest health benefits. Furthermore, evidence suggests that physical inactivity and sedentary behavior tend to cluster with other modifiable health risk factors, including poor dietary intake, insufficient sleep and substance abuse [15, 16]. Identifying characteristics of individuals with unique activity-related behavioral typologies could help strengthen interventions promoting healthy behaviors and in addition, help to further refine and target strategies among those most in need.

Ecological models help to frame or explain the complexity of factors that influence youth health behaviors and posit that a combination of factors across multiple levels (e.g., intrapersonal, interpersonal, environmental) interact to influence an individual’s behavior [17]. Some factors are more modifiable (amenable to change) than others (e.g., physical activity self-efficacy versus age or sex). While there is an abundance of literature identifying ecological correlates of participation in individual activity-related behaviors (e.g., physical activity *or* sedentary behavior), an understanding of the potential influences on *combinations* of these behaviors among youth is limited. Ferrar et al. [9] published a systematic review of 19 studies that identified clusters of time-use behaviors among adolescents, and the modifiable correlates associated with these. However, it included only studies published up to 2010 and a diverse range of activities of daily living (e.g., socializing, grooming and chores). Furthermore, studies that considered lifestyle factors such as diet and risk-taking behaviors (e.g., smoking) were excluded [9]. More recently, Leech et al. [18] reviewed studies published up to November 2012 and identified typologies based on physical activity, sedentary behavior and diet among children and adolescents and differences according to age, sex, socioeconomic status (SES) and weight status. Modifiable correlates (e.g., self-efficacy, parental support for physical activity) of typology membership were not considered in their review [18].

Interventions targeting multiple behaviors have the potential to elicit reciprocally favorable benefits. Moreover, identifying the key sociodemographic characteristics and modifiable correlates associated with different typologies of physical activity and sedentary behavior is imperative for gaining insights into potential behavior change strategies for those most at risk of engaging in unhealthy behavior typologies. Sociodemographic characteristics describe the composition of individuals within a typology and inform who to target, while modifiable correlates inform what to target. This systematic review aimed to synthesize the socio-demographic characteristics and modifiable correlates of activity-related behavioral typologies among adolescents.

## Methods

This review was registered with PROSPERO (registration number: CRD42016046879) in September 2016 and followed the Preferred Reporting Items for Systematic Review and Meta-Analysis (PRISMA) guidelines [19].

### Search strategy

Papers published online up to and including April 2017 were identified through seven electronic databases: CINAHL complete, Medline complete, Psychology and Behavioral Sciences Collection, PsychINFO, SPORTDiscus, Web of Science and ProQuest. This search was then updated at the end of May 2018. Four key search strings were searched using the AND operator (‘typology’, ‘physical activity’, ‘sedentary behavior’ and ‘adolescents’). Terms within each search string were separated by the OR operator (see Additional file 1 for the list of terms).

### Selection criteria and screening

The criteria used to determine eligibility of each article for inclusion were 1. be a quantitative study; 2. include adolescents with an average sample age between 12 and 18 years; 3. be a peer-reviewed original research articles, have human participants and be written in English language; 4. include a measure of both physical activity and sedentary behavior (to reflect the activity spectrum); and 5. have undertaken a person-oriented statistical approach to identify typologies.

**Fig. 1:**
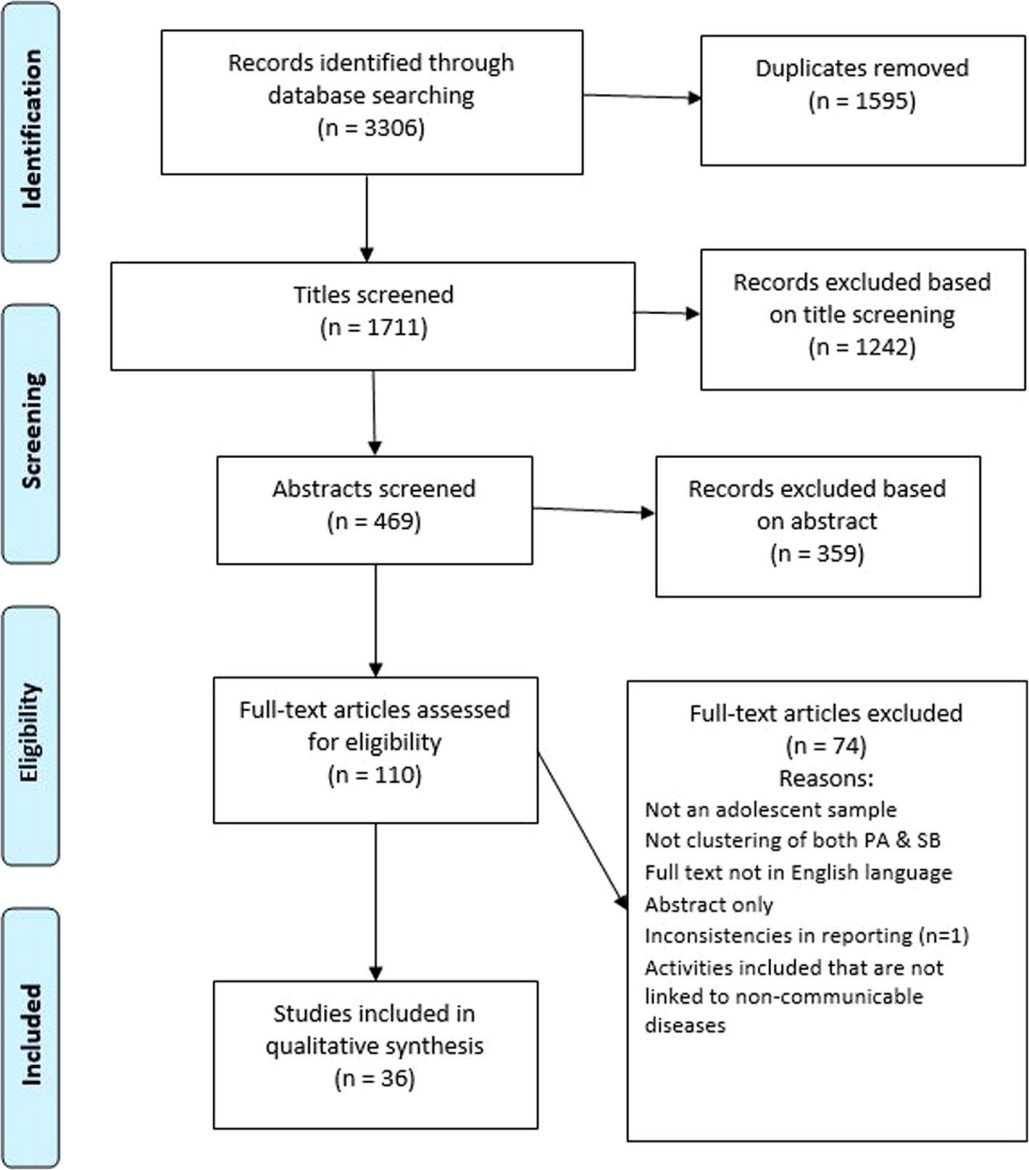
Flow of study inclusion for the review

The initial search across the seven databases yielded 3306 results, which after removal of duplicates was reduced to 1711. Following title screening (1711 papers) by KP, all further screening was conducted independently by two reviewers (KP and SC). Title screening resulted in 469 papers eligible for abstract screening. Following abstract screening, 110 papers were read in full and any discordance over papers to include was discussed and an outcome agreed upon by the team. All included papers (*n* = 36) then underwent risk of bias and methodological quality assessment and data extraction. Figure 1 shows the flow of study inclusion for the systematic review.

### Data extraction and quality assessment

Risk of bias and methodological quality of included studies was assessed using a modified tool adapted from two quality assessment tools [20, 21] tailored towards observational and longitudinal research [20, 21]. Full details of the adapted methodological quality tool have been reported elsewhere [22]. In brief, the modified tool assesses 15 methodological components of observational research studies including: study design (e.g., cross-sectional, longitudinal), selection bias (e.g., sampling, response rate, and representativeness), confounders (e.g., controlling for confounders such as age, sex etc.), data collection methods (e.g. valid, reliable), and withdrawals and dropouts (e.g., % complete data). Each component was assigned either a zero (not described or referred to) or a half/one (adequately described or referred to) to provide a methodological quality score out of a total of 15. Studies were considered to be of high quality if they scored greater than 50% (7.5/15) [22]. Two independent reviewers (KP and SC) assessed the quality of all studies. There was good inter-rater reliability (Pearson *r* = 0.79, *p* < 0.01) for assessing study quality between reviewers. The most common discrepancies between the reviewers were for the numbers of participants at each stage, participant response rates, and how missing data was dealt with. All discrepancies across the components were discussed and resolved by consensus by KP and SC.

## Results

A total of 36 articles published from 2002 to 2018 were included in the final systematic review. For ease of interpretation, three sub themes were used to help classify the typologies. These included studies that based typologies on 1) physical activity and sedentary behavior measures only, 2) risk-related behaviors (e.g., substance abuse and smoking) in addition to physical activity and sedentary behavior, and 3) diet-related factors in addition to physical activity and sedentary behavior.

### Study characteristics

Additional file 2 provides an overview of the descriptive characteristics and methods employed within each study. In brief, the majority were cross-sectional studies (*n* = 27), with the remaining nine being longitudinal. Sample sizes ranged from *n* = 195 [23] to *n* = 109,104 [24, 25] with most including a relatively even distribution of males and females. Participant ages differed between study samples with the largest age range being 3–18 years [26].

Most of the studies assessed physical activity using subjective methods such as self-report surveys (*n* = 29) [24–51], checklists (*n* = 1) [52] and interviewer administered questionnaires (*n* = 3) [53–55]. Approximately half of these studies (*n* = 17, 52%) reported validity and/or reliability of the measures. Three studies measured physical activity objectively, including accelerometers to measure activity intensity [56], activPAL devices to measure step and activity counts [23], and one used both subjective (self-report survey) and objective (accelerometer) measures to assess physical activity participation [12]. For sedentary behavior assessment, 94% of studies used subjective methods, including self-report surveys (*n* = 30) [12, 24–51], checklists (*n* = 1) [52] and interviewer administered questionnaires (*n* = 3) [53–55]. Of these, 35% (*n* = 12) indicated validity and/or reliability. A variety of sedentary behaviors were assessed within these subjective measures. Most included only screen time variables (*n* = 22) [13, 26–30, 32, 34, 36–41, 43, 44, 47–50, 53, 54]. Four studies assessed screen-based and educational sedentary behaviors as separate variables [31, 35, 45, 55], with three additionally assessing social sedentary behaviors (e.g., talking on phone) [12, 51, 52]. Four studies included a summary measure combining screen-based, educational and social sedentary behaviors [24, 33, 42, 46]. Just one study included a measure of passive travel in addition to screen-based sedentary behavior [37], and one asked participants to self-report their time spent sitting in general [25]. Only two studies measured sedentary time objectively using accelerometers [56] or activPAL devices [23].

With the exception of weight status (determined in five studies by researcher-measured height and weight), correlates were measured using self-report methods across all studies. Validity and/or reliability of survey items were not reported by any studies that assessed modifiable correlates. The statistical approach to identify the activity-related behavioral typologies most commonly used by studies included in this review (see online Table 1) was cluster analysis (*n* = 18), followed by latent class analysis (*n* = 13), latent profile analysis (*n* = 2), principal component analysis (*n* = 1), exploratory and confirmatory factor analysis (*n* = 1), and self-organizing maps analysis (*n* = 1).

### Descriptive characteristics of behavioral typologies

Thirteen studies (36%) based activity-related behavioral typologies on combinations of physical activity and sedentary behavior only. The most commonly reported behavioral typology based only on these behaviors (Table 1) was that of high physical activity and low sedentary behavior (*n* = 11) [12, 23, 30, 35, 36, 45, 48, 51, 53, 54], comprised mainly of adolescents under 15 years. Other common behavioral typologies were those characterized by low physical activity and high sedentary behavior (*n* = 8) [23, 35, 36, 45, 48, 49, 53] with higher proportion of overweight/obese adolescents, high physical activity and high sedentary behavior engagement (*n* = 5) [13, 30, 36, 51, 52] and low engagement in all behaviors (*n* = 10) [12, 35, 36, 48, 49, 51, 52, 54]. Table 1 provides an overview of the descriptive characteristics of these typologies.

**Table 1:** Typologies identified by physical activity and sedentary behavior only

	High PA/High SB	High PA/Mod SB	High PA/Low SB	Mod PA/High SB	Mod PA/Mod SB	Mod PA/Low SB	Low PA/High SB	Low PA/Mod SB	Low PA/Low SB
Socio-demographic characteristics									
Age	+ (36) 0♂ (53)	+♂ (49) 0♂ (53)	+♀ (23) - (36, 37, 54, 55) -♂ (12) 0♀ (12)			0♀ (12)	+ (49) 0♀ (12)		+ (37, 54, 55) +♀ (53) -♂ (49) 0♂ (53)
Sex (male)	+ (36, 37, 52)		+ (54) - (52) 0 (46)		- (52)		+ (54) 0 (46)		- (37, 54) 0 (46)
Weight status	0 (53) + (30, 36)	0 (53)	-♀ (23) - (12, 54)				+ (12, 36, 54) +♀ (23)	+ (30)	- (54) 0 (53) + (36)
Household income			+ (55)	- (55)					
Family affluence			0 (12) + (49)			0 (12)	0 (12)		+ (49)
Ethnicity (white)			+ (55) 0♂ (12)	- (55)		0♂ (12)	0♂ (12) -♀ (12)		
Ethnicity (Hispanic)	0♂ (36) -♀ (36)		-♀ (55) 0♂ (36, 55)	0♂ (55)		0♂ (55)	0♂ (36)		0♂ (36, 55) +♀ (36, 55)
Ethnicity (Black)			- (55)	- (55)					
Ethnicity (Asian)			0 (55)	0 (55)		0 (55)			0 (55)
Ethnicity (Not specified)	0 (53)	0 (53)							0 (53)
Country of birth (born in study country)	0♂ (53)	0♂ (53)		+ (55)					- (55) 0♂ (53) +♀ (53)
Education level	- (52)		- (46) -♂ (12) 0♀ (12)			0♀ (12)	0♀ (12) + (46, 52)		
Live with 2 parents			0♂ (12)			0♂ (12)	0♂ (12) -♀ (12)		
Parental education level			0 (12) 0 (54)			0 (12)	0 (12, 54)		0 (54)
Behavioral correlates									
School attendance			+♂ (55) -♀ (55) +♀(low skating/biking) (55)	-♀ (55)		+♀ (55)			- (55)
Academic performance	+ (35)		+ (35)		+ (35)				

♂ indicates male only, ♀ indicates female only, + indicates positive association, − indicates negative association, 0 indicates no association, PA; physical activity, SB; sedentary behavior

Ten studies based typologies on physical activities, sedentary behaviors and risk-related behaviors (28%). These studies consistently found a typology defined by optimal levels of physical activity and sedentary behavior, along with low risk-related behaviors [24, 28, 29, 32, 33, 38, 39, 42]. A typology that was characterized by high risk-related behaviors was found in all 10 studies [24, 28, 29, 32, 33, 37–40, 42], typically comprised of older adolescents. Three of these studies additionally found a typology characterized by high risk behaviors combined with sufficient activity levels [33, 40, 42]. Table 2 describes the descriptive characteristics of these typologies.

**Table 2:** Typologies identified by physical activities, sedentary behaviors and health risk-related behaviors

	Healthy activity, low risk	Average activity, low risk	Low activity, low risk	Sedentary, low risk	Healthy activity, average risk	Average activity, average risk	Low activity, average risk	Sedentary, average risk	Healthy activity, high risk	Average activity, high risk	Low activity, high risk	Sedentary, high risk
Descriptive characteristics												
Age	-♂ (28) - (24)	-♂ (28) - (32) +♀ (28)	+♀ (28) + (41)	+♂ (32)					- (41)	+♀ (28) +♂ (32) + (38, 40)	+♀ (32) + (24)	+ (29)
Sex (male)	- (24, 29)				- (38)	+ (29)		- (24)	+ (41)	- (41)	+ (24)	
Weight status	- (32, 39, 40)			+♀ (28) + (39)	+ (39)			+♂ (28)		+♀ (28) + (38–40)		+ (29)
Household income	+♀ (28)			-♀ (28)	+♂ (28)		-♂ (28)	-♂ (28)			-♂ (28)	
Family affluence				- (29)		+ (29)						
Parental education level	+♀ (28) + (24)		-♀ (28)	-♀ (28)	+♂ (28)		-♂ (28)	-♂ (28) + (24)		-♀ (28)		
Ethnicity (white)	+ (40)			-♀ (28)	+♀ (28)			-♂ (28)				
Ethnicity (Hispanic)	-♀ (28)	+♀ (28)			-♂ (28)	+♀ (28)	+♂ (28)	+♂ (28)			+♂ (28)	
Ethnicity (Black)	-♂ (28)			+♀ (28)				+♂ (28)				
Ethnicity (Asian)		+♀ (32)										-♀ (32)
Ethnicity (Multiethnic)		+♀ (28)				+♀ (28)						
Country of birth (born in study country)	+ (29)			- (29)		+ (29)				+ (38)		
School type (public)											+ (24)	
Education level					+ (38)							
Individual correlates												
Self-efficacy												- (29)

♂ indicates male only, ♀ indicates female only, + indicates positive association, − indicates negative association, 0 indicates no association

The remaining 13 studies based typologies on physical activities, sedentary behaviors and diet-related factors (36%). Eight of the 13 studies found a typology of adolescents characterized as active with a healthy diet [27, 31, 34, 43, 44, 50, 55, 56], and three found a typology characterized by ‘average’ levels of physical activity and sedentary behaviors combined with a healthy diet [35, 41, 43]. Adolescents in active typologies, regardless of diet quality, were typically younger [31, 34, 43, 44, 46, 56] and of healthy weight status [26, 27, 47]. Typologies that were defined by high screen time were typically also characterized by unhealthy diet quality indicators [31, 34, 44, 47, 50], however two studies reported a typology characterized by high screen time and an average diet [27, 43]. These studies were generally female dominated with a larger proportion of older adolescents. Descriptive characteristics of these typologies are provided in Table 3.

**Table 3:** Typologies identified by physical activities, sedentary behaviors and diet-related factors

	Active, healthy diet	Active, average diet	Active, unhealthy diet	Average activity, healthy diet	Average activity, average diet	Inactive, healthy diet	Inactive, average diet	Inactive, unhealthy diet	High screen, healthy diet	High screen, average diet	High screen, unhealthy diet	Sedentary, healthy diet	Sedentary, average diet	Sedentary, unhealthy diet
Descriptive characteristics														
Age	- (31, 34, 45) -♂ (57) 0 (56) 0♀ (57)	- (47) - (44)	- (31, 47)			0♀ (57)	+ (34, 51) - (42)	0 (56) + (44)			+♂ (31) - (51)	+♀ (31) +♂ (57)		0♀ (57)
Sex (male)	+ (31, 56) - (34)	+ (42)	+ (31)	+ (47)	+ (25, 48)	- (47, 48)	+ (34) - (42, 51)	- (48, 56)		+ (27)	+ (48, 51)	- (31, 42)		- (25)
Weight status	- (27) 0 (56) 0♀ (57)	0 (47)	0 (47)	- (26) + (42)	- (26, 48)	0 (47) 0♀ (57))		0 (47, 56) - (48) - (44)		+ (27)	+ (48) - (51)	+♂ (57)	0 (47)	0♀ (57) + (42)
Neighborhood deprivation		- (42)												+ (42)
Household income														
Family affluence	+ (27, 34) 0 (56)	0 (47)	0 (47)		+ (48)	0 (47) + (48)	- (27, 34)	0 (47, 56) + (44)		- (27) - (44)	- (34, 48)		0 (47)	
Parental education level	0 (56)	+ (47)			- (25)	+ (47)		0 (56)					+♂ (47)	+ (25)
Ethnicity (white)	+ (34)					+♀ (57)		+♂ (57)			- (34)			- (42)
Ethnicity (Hispanic)	- (27) -♀ (34) +♀ (57)						+ (27) +♀ (34)							
Ethnicity (Black)	- (27) -♀ (34) +♂ (57)				- (25)		+ (27) +♀ (34)			+ (27)				+♀ (57) + (25)
Ethnicity (Asian)	+ (27)						- (27)							+ (42)
Country of birth (born in study country)				+ (42)							- (48)			
Urbanicity (capital city)					0 (25)									0 (25)
Individual correlates														
Self-regulation							- (51)							
Self-concept	+ (27)													
Motivation	+ (51)						- (51)				- (51)			
Body satisfaction							- (51)							
Perceived health		+ (42)					- (51)					+ (42)		- (42)
Behavioral correlates														
School engagement	+ (27, 51)						- (51)		- (27)		- (51)			
Educational aspiration	+ (45)													
Social correlates														
Relationship with parents							- (51)							
Relationship with teachers	+ (27)								- (27)					
Relationship with classmates	+ (27)						- (51)		- (27)					

♂ indicates male only, ♀ indicates female only, + indicates positive association, − indicates negative association, 0 indicates no association

### Modifiable correlates of typologies

Of the 36 studies included in this review, only eight reported modifiable correlates associated with typology membership. Of these, seven assessed intrapersonal or behavioral factors (e.g., school attendance, self-esteem, motivation) [27, 29, 35, 41, 44, 50, 54], two studies assessed interpersonal factors (e.g., parental support and interaction) [27, 50], and none explored environmental factors. Each of these studies were cross-sectional.

#### Correlates of typologies identified by physical activity and sedentary behavior only

Only two studies that identified typologies based on physical activity and sedentary behavior explored modifiable correlates (Table 1). Adolescents who attended school [54] and had good academic performance (as indicated by a grade point average ≥ 8.5) [35], were more likely to be in typologies characterized by moderate or high physical activity levels, regardless of sedentary behavior, compared to typologies with low physical activity levels. No other intrapersonal correlates, nor interpersonal or environmental correlates, were assessed.

#### Correlates of typologies identified by physical activities, sedentary behaviors and risk-related behaviors

Self-efficacy was the only modifiable correlate assessed in relation to typologies based on physical activities, sedentary behaviors and risk-related behaviors (Table 2) [29]. Adolescents within the typology characterized as sedentary and engaging in high risk-related behaviors had significantly lower general self-efficacy [29] compared to those in the other six unique typologies.

#### Correlates of typologies identified by physical activities, sedentary behaviors and diet-related factors

At the intrapersonal level (Table 3), adolescents who were more engaged in school [27, 50] and had greater educational aspirations were more likely to be in a typology characterized by high levels of physical activity and a healthy diet compared to typologies characterized by inactivity or engaging in high screen time and consuming less healthy diets [44]. Berlin et al. [27] found adolescents within this typology reported significantly higher levels of self-concept than adolescents in typologies characterized by inactivity or high screen time and an average diet. Veloso et al. [50] found that adolescents within the typology characterized as ‘inactive and average diet’ had significantly lower perceived health, self-regulation and body satisfaction when compared to those characterized as ‘active with a healthy diet’. Similarly, Mandic et al. [41] found that adolescents in a typology characterized by sedentariness and an unhealthy diet had significantly lower perceived health compared to those in typologies characterized by more activity or a healthier diet. Conversely, motivation for physical activity was found to be significantly higher among adolescents in typologies characterized by high physical activity compared to those in typologies characterized by ‘inactivity and an average diet’, or ‘high screen time with an unhealthy diet’ [50].

At the interpersonal level, adolescents who reported strong relationships with their parents [50], teachers [27] and classmates [27, 50] were less likely to be in typologies characterized as ‘inactive and consume an average diet’, or ‘high screen time and an unhealthy diet’ when compared to adolescents who were ‘active and consume a healthy diet’.

### Risk of bias and methodological quality

The average method quality score was 9.8/15 with no systematic differences in findings between studies according to study quality. Out of the 36 included studies, 30 were considered to be of high quality with a score greater than 7.5/15. The most common methodological components that were missing from the studies reviewed included reliability and validity of the self-report measures of physical activity and/or sedentary behavior, numbers of participants at each stage of the study (e.g., eligible, consented, provided data) and numbers of participants with missing data for physical activity and sedentary behavior.

## Discussion

This systematic review synthesized findings from 36 studies to determine activity-related behavioral typologies of adolescents, and associated sociodemographic characteristics and modifiable correlates. While difficult to compare studies due to the range of behaviors, study samples and data analysis, there was some consistency in typologies and their characteristics across studies. Consistent results were found with typologies characterized by high physical activity combined with low sedentary behavior, and low physical activity combined with high sedentary behavior being common. The low physical activity and high sedentary behavior typology often co-occurred with high risk-related behaviors or poor diet quality. Socio-demographics characteristics were assessed across most of the 36 included studies; however, modifiable correlates were assessed scarcely suggesting further research in this area is required.

Typologies characterized by unhealthy behavior patterns appeared to be consistently comprised of predominantly older adolescents. It is unknown, however, whether younger adolescents who engage in high levels of physical activity and low sedentary behavior continue this health promoting combination as they age. To date, only one activity-related typology study employed a longitudinal design assessing long term physical activity and sedentary behavior engagement dependent on baseline typologies, however, that study examined independent changes in physical activity and sedentary behavior into young adulthood [54]. Recently, researchers have begun utilizing growth trajectory modelling to assess developmental trajectories of health behaviors. However, no studies have explored the trajectories of physical activity and sedentary behavior typologies during childhood or adolescence. Future studies should assess long term maintenance, or trajectories, of physical activity and sedentary behavior based on typology membership. In general, there were no consistent differences in typology membership for boys and girls regardless of the typology.

There was limited consistency in ethnicity, socio-economic status or household composition between typologies due to large variations between study samples. Weight status was consistently found to be higher among adolescents in typologies characterized by risk-related behavior patterns [28, 29, 37, 38], largely attributable to low physical activity levels [12, 13, 23, 53]. Additionally, healthy weight status tended to be more common among adolescents in more active typologies [26, 27, 32, 38, 39, 47]. These findings highlight the need to find ways to increase active behaviors throughout adolescence, while also considering other modifiable health risk factors that tend to cluster with physical inactivity (e.g. risk-related behaviors and unhealthy diet).

Very few studies included in this review examined modifiable correlates of activity-related typologies, especially at the environmental level. In general, better scores or levels of psychological and academic-related factors tended to be associated with typologies characterized by high levels of physical activity, in many cases regardless of other co-occurring behaviors included in the typologies. For example, adolescent typologies characterized by high levels of physical activity with a healthy diet [27] had higher self-concept and self-esteem than typologies characterized by lower levels of activity, emphasizing the importance of physical activity for emotional wellbeing. Engagement in high levels of physical activity, regardless of other co-occurring behaviors was positively associated with school attendance [54], engagement [27, 50], academic performance [35, 57], and education aspiration [44]. The consistency in these findings highlight the need for strategies targeting an increase in adolescent physical activity levels in particular.

Only two studies assessed interpersonal correlates, with results suggesting that relationships with others are positively associated with typologies characterized by a high level of physical activity [27, 50]. The lack of interpersonal correlates assessed is surprising given that social interaction and support are widely accepted correlates of activity-related behaviors. For example, parent and family support are key correlates of physical activity [58, 59] and sport participation [60], while friend and peer social interactions and norms are more strongly associated with active travel [61]. In regard to sedentary behavior, parental modelling and parental rules are key predictors of screen-time specifically [62]. Similarly, environmental correlates of typologies have largely been overlooked, with only urbanicity (capital city vs. not capital city) being considered [25]. Evidence has shown that adolescents living in neighborhoods perceived to be more conducive to physical activity typically participate in more physical activity [63–65] and less sedentary behaviors [62]. Based on current evidence, it is difficult to identify consistent correlates of activity-related typologies. Few have explored modifiable correlates. Further, all studies that examined modifiable correlates included in this review were cross-sectional and therefore the directionality of the results is unclear. For example, it is unclear if high self-concept determines typology membership or vice versa. Studies examining typologies should consider identifying modifiable correlates of typologies to better inform intervention strategies. In addition, longitudinal research is needed to examine stability of typologies and determinants of typologies over time.

The methodological quality assessment revealed that most (30 out of 36) studies were of high quality [22]. Overall, reporting of sample selection and recruitment, measures included and appropriate statistical analysis methods were thorough and clear with no systematic differences in findings between studies according to study quality. However, the majority of studies utilized subjective measures of physical activity and sedentary behavior to determine typologies. The limited reporting of validity or reliability of these measures made it difficult to determine the robustness of the typologies identified. Future research needs to ensure that validity and reliability of activity-related behavior measures are reported. Although cluster analysis has been used repeatedly in typology research, there was wide variation in the cluster algorithms used to generate the clusters across the studies included in this review (i.e., Ward’s, K-means, or two-step) and this type of analysis largely relies on subjective decision-making around typology identification (e.g., number of clusters, size of clusters) [66]. Although less frequently used, it has been suggested that latent class analysis, latent profile analysis, principal component analysis and self-organizing maps analysis, are more reliable methods than cluster analysis due to greater reliability of statistical measures to identify the typology solution [66]. It should also be noted that in many cases, typologies reflect ‘higher’ or ‘lower’ levels of a behavior relative to others in the study sample, rather than optimal or below-optimal levels (i.e., meeting the recommended physical activity levels or not).

The benefits of physical activity, including active travel, sport and leisure-time active play, are well-known [67] and the negative health impacts of some sedentary behaviors, such as screen-time, are emerging [3]. The current review focused a-priori on typologies that included behaviors across the activity spectrum (i.e., both physical activity and sedentary behaviors). However, most of these studies were limited to screen time and did not include a range of sedentary behaviors. In addition, some of these studies also included diet or risk behaviors in determining typologies. It should be noted that it is currently not clear what combination of behaviors is most ideal. A previous review, for example, found inconclusive evidence that cluster patterns based on physical activity, sedentary behavior and diet were associated with overweight and obesity [18]. It is possible that within a given typology, one specific behavior may drive associations with health outcomes, or that behaviors other than physical activity or sedentary may be more important. Further research should be undertaken to establish the synergistic impact of activity-related and other behaviors on health and wellbeing. In addition, while the inclusion of different behaviors across studies made the typologies identified difficult to compare, the data-driven techniques used also give rise to the possibility that the typologies are unique to each study sample. It is possible that even if the same behaviors (and measures) were included across studies, very different typologies could emerge using such data-driven approaches.

## Conclusions

This systematic review included studies published between 2002 and 2018, and typologies based on a wider range of health-related behaviors than has previously been reviewed. Despite heterogeneity, it is evident that activity-related behaviors cluster in different ways in specific groups of adolescents. While further research is needed, tailoring strategies for unique groups in future interventions should be considered, rather than ‘one size fits all’ approaches. To do this, more evidence on modifiable correlates of different typologies of physical activity and sedentary behavior is required. With the exception of descriptive characteristics defining who makes up the typologies, very few modifiable correlates have been studied to date. Investigation into the added worth of focusing on multiple versus single behaviors in health promotion is needed. However for sustained behavior change, it is suggested that interventions should utilize multi-component approaches [68]. Therefore, future research needs to assess a more thorough range of modifiable correlates associated with activity-related behavior typologies.

## Additional files

Additional file 1: Search terms. This file includes a table providing an overview of the search terms and strings that were used to identify the literature for the review. (DOCX 12 kb)

Additional file 2: Study details. The file includes a large table that provides the details of all included studies. (DOCX 76 kb)
