# Supplementary material for: Activity-related behavior typologies in youth: a systematic review
Source: Int J Behav Nutr Phys Act. 2019 May 16;16:44. doi: 10.1186/s12966-019-0804-7 (PMC6524235; doi:10.1186/s12966-019-0804-7)
Supplement: Supplementary file 1 — Search terms. This file includes a table providing an overview of the search terms and strings that were used to identify the literature for the review. (DOCX 12 kb) [file 12966_2019_804_MOESM1_ESM.docx]

**Online table 1. Search terms**

| **Search term** | **Search string** |
| --- | --- |
| Statistical approach | “Typology” OR “cluster” OR “latent class” OR “latent profile” OR LCA OR LPA OR “behaviour pattern” OR “lifestyle pattern” |
| Physical activity | (“physical activity” OR “exercise” OR “active travel” OR “sport” OR “recreation” OR “active play” OR “commute” OR “walk” |
| Sedentary behaviour | (“sedentary behaviour” OR “sedentary” OR “sitting” OR “screen” OR “TV” OR “television” OR “computer use” OR “sit” OR “TV view” OR “video gaming” OR “electronic gaming” OR “e-gaming” OR “computer gaming” |
| Adolescents | “adolescent” OR “youth” OR “teen” OR “secondary school” OR “high school” |
