# Supplementary material for: Activity-related behavior typologies in youth: a systematic review
Source: Int J Behav Nutr Phys Act. 2019 May 16;16:44. doi: 10.1186/s12966-019-0804-7 (PMC6524235; doi:10.1186/s12966-019-0804-7)
Supplement: Supplementary file 2 — Study details. The file includes a large table that provides the details of all included studies. (DOCX 76 kb) [file 12966_2019_804_MOESM2_ESM.docx]

**Online table 2. Study details**

|  |  | Typology analysis | | | | | Results | |  |
| --- | --- | --- | --- | --- | --- | --- | --- | --- | --- |
| First author  Year  Country | Participant characteristics | Analytical technique used | Physical activity measure | Sedentary behavior measure | Additional behaviors | Socio-demographic variables/correlates examined | Typologies identified in papers | Typologies defined by review authors | Method quality score  (%) |
| (24)Azeredo  2016  Brazil | 13-16 years  N=109,104  47.8% male | Exploratory and confirmatory factor analysis | PA | 'Sedentarism' (=combination of)  TV  Computer  Video games  Talking to friends  Other activities apart from school | Bullying; Physical fight; Fight with gun; Fight with weapon; Smoking; Drinking alcohol; Glasses of alcohol; Drug use; Number of sexual partners; Unsafe sex; Bagged salty snacks; Fried salty snacks; Fizzy drinks; Raw vegetable intake; Cooked vegetable intake; Fruit intake | Age; Sex; Geographic region; School admin status | 1. Problem-behavior  2. Health-compromising diet and sedentary behavior  3. Health-promoting diet and physical activity behavior | 1. Low activity, high risk  2. Sedentary, average risk  3. Healthy activity, low risk | 63.3% |
| (27)Berlin  2017  United States | Mean age=14.1±0.4  n=9295  51.6% male | Latent profile analysis | School sports  Non-school sports  Days of exercise  Days of PE | TV/video/DVD  Video/computer games  Internet use | Diet | Age; Ethnicity; Weight status; Sex; SES; Internalizing problems; Self-concept; Locus of control; Fit in at school; Close to classmates; Close to teachers; Enjoy school | 1. Active & healthy diet (AHD)  2. Sedentary & unbalanced diet (SUD)  3. Screen-time & junk food (STRF) | 1. Active, healthy diet  2. Inactive, average diet  3. High screen, average diet | 60% |
| (28)Boon-Heinonen 2008  United States^a^ | 11-19 years  n= 8840 49.3% male | Cluster analysis | Skating Sport Exercise School team sport School individual sport PE | TV  Videos Computer/video games | Diet  Risk-related behaviors | Ethnicity; Parental education; Household income; Age; Weight status | Males  1. School clubs & sports  2. Sports  3. Moderately active  4. Sedentary behaviors  5. Funk food & smoke  6. Dieters  7. Low diet & activity  Females  1. School clubs & sports  2. Average diet & activity  3. High consumer  4. Sedentary behaviors  5. Junk food, low activity  6. Restrictive dieting & smoke | Males  1. Healthy activity, average risk  2. Healthy activity, low risk  3. Average activity, low risk  4. Sedentary, average risk  5. Low activity, high risk  6. Healthy activity, low risk (except dieting)  7. Low activity, average risk  Girls  1. Healthy activity, low risk  2. Average activity, low risk  3. Average activity, average risk  4. Sedentary, low risk  5. Low activity, low risk  6. Average activity, high risk | 63.3% |
| (29)Busch  2013  Netherlands | Mean age=14  n= 2690  45% male | Principal component analysis  AND  Two-step cluster analysis | Physical exercise (including walk, bike, gym class, sports) | TV  Internet use Videogames | Cannabis use; Alcohol use; Binge drinking; Smoking; Nutrition; Compulsive internet use; Compulsive videogames;  Being a bully; Bully victim | Age; Sex; SES; Ethnicity; BMI; Education level; General self-efficacy | 1. Healthy 2. 2nd healthiest overall/problematic screen time 3. Most unhealthy behavioral patterns 4. 3 healthiest overall/unhealthy sedentary behavior | 1. Healthy activity, low risk  2. Average activity, average risk  3. Sedentary, high risk  4. Sedentary, low risk | 46.7% |
| (30)Carson  2015  Canada | Mean age=15.7  n= 19,831  48.7% male | Latent class analysis | MPA^b^  VPA^b^ | TV viewing^b^  Internet use^b^  Video/computer games^b^ | Sleep | Weight status^b^ | 1. Healthiest movers  2. Active screenies  3. Unhealthiest movers | 1. High/Low  2. High/High  3. Low/Mod | 80% |
| (31)Cuenca-Garcia 2013  Europe | Mean age=14.8±1.2  n= 2084  45.6% male | Two-step cluster analysis | MVPA^b^ | Homework^b^ Screen time^b^ | Diet quality | Age; Sex | 1. Healthy diet and active 2. Healthy diet and academic 3. Healthy diet and inactive 4. Unhealthy diet and screen user 5. Unhealthy diet and active | 1. Active, healthy diet  2. Sedentary, healthy diet  3. Inactive, healthy diet  4. High screen, unhealthy diet  5. Active, unhealthy diet | 66.7% |
| (23)Dowd  2016  Ireland | Mean age=15.7±0.9  n= 195  100% female | Cluster analysis with Ward’s method with squared Euclidean distances | MVPA^b^ | Sitting/lying time^b^ | Standing time; LIPA | Age | 1. Low  2. Moderate  3. High | 1. Low/High  2. Mod/Mod  3. High/Low | 93.3% |
| (32)Fleary  2017  United States | 9-12^th^ grade  n= 14,815  51% male | Latent class analysis | MVPA | Total sedentary including:  TV  Video games  Computer for leisure | Smoking; Alcohol; Binge-drink; Marijuana use; Physical fight; Insufficient sleep; Unhealthy weight control; Tried to lose weight; Fruits 2+ days; Veg 2+ days; F&V 5+ days; Breakfast daily | Age;  Perceived overweight status; BMI | Boys  1. Healthy  2. Sedentary  3. Physically active  4. Health risk behaviors/PA  Girls  1. Healthy  2. Physically active  3. Sedentary  4. Health risk behaviors/F&V  5. Health risk behaviors | Boys  1. Average activity, low risk  2. Sedentary, low risk  3. Healthy activity, low risk  4. Average activity, high risk  Girls  1. Average activity, low risk  2. Healthy activity, low risk  3. Sedentary, low risk  4. Sedentary, high risk  5. Low activity, high risk | 53.3% |
| (57)Hartz  2018  United States | Mean age=15.4±0.7  n=1233  51.5% male | Latent class analysis | MVPA^b^ | Sedentary time^b^ | Diet quality | BMI^b^; Age; Ethnicity | Boys   1. Healthy diet-Healthy PA-Healthy ST 2. Unhealthy diet-Unhealthy PA-Healthy ST 3. Healthy diet-Unhealthy PA-Unhealthy ST | Boys   1. Active, healthy diet 2. Inactive, unhealthy diet 3. Sedentary, healthy diet   Girls   1. Active, healthy diet 2. Inactive, healthy diet 3. Sedentary, unhealthy diet | 80% |
| (33)Heikkala  2014  Finland | 16 years old  n=6892  47.9% male | Latent class analysis | MVPA | Sedentary activity including:  TV  Reading books/magazines  Computer  'Other' sedentary activities | Externalizing problems; Internalizing problems; Sleeping; Smoking; BMI |  | Boys  1. Externalizing behavior  2. Sedentary  3. Obese  4. Reference (high PA, sleep/ low internalizing, sb, bmi, smoking)  Girls  1.Externalizing behavior  2. Multiple risk behaviors  3.Obese  4. Reference (high sleep, pa/ low smoking, bmi, internalizing) | Boys  1. Average activity, high risk  2. Sedentary, Low risk  3. Average activity, low risk  4. Healthy activity, low risk  Girls  1. Healthy activity, high risk  2. Sedentary, high risk  3. Low activity, average risk  4. Healthy activity, low risk | 66.7% |
| (34)Iannotti  2013  United States | Mean age=13.9  n= 9227  48.4% male | Latent class analysis | PA^b^ | TV^b^ Video or computer games^b^ Computer^b^ | Fruits; Vegetables; Sweets; Sweetened soft drink; Chips; French fries | Sex; Age; Ethnicity; Family affluence | 1. Healthful 2. Unhealthful 3. Typical | 1. Active, healthy diet  2. High screen, unhealthy diet  3. Inactive, average diet | 70% |
| (35)Kantomaa  2015  Finland | 15-16 years old  n= 12,081  51.2% male | Latent class analysis | LTPA^b^  Sport^b^ | TV  Reading books or magazines  Computer and video games  Other sedentary activities | Sleep | Grade Point Average^b^ | 1. Sedentary TV viewers  2. Generally inactive  3. Moderately active readers  4. Active sport clubbers  5. Generally active | 1. Low/High  2. Low/Low  3. Mod/Mod  4. High/Mod  5. High/Low | 70% |
| (36)Kim  2016  United States^a^ | 9-12^th^ grade  n= 12081  50.6% male | Latent class analysis | MVPA Team sport Muscle strengthening exercise | TV Computer for leisure |  | School grade; Ethnicity | 1. High PA/Low SB  2. High PA/High SB  3. Low PA/High SB  4. Low PA/Low SB | 1. High PA/High SB  2. High PA/Low SB  3. Low PA/High SB  4. Low PA/Low SB | 80% |
| (37)Kim  2016  United States^a^ | 9-12^th^ grade  n= 18253  49.5% male | Latent class analysis | MVPA Team sport Muscle strengthening exercise | TV Computer for leisure |  | Sex; School grade | 1. High PA/Low SB  2. High PA/High SB  3. Low PA/High SB  4. Low PA/Low SB | 1. High PA/LowSB  2. High PA/High SB  3. Low PA/High SB  4. Low PA/Low SB | 36.7% |
| (26)Kontogianni  2010  Greece | 3-18 years old  Mean age children=7.6±2.9, adolescent=15.5±1.6  n=1305  Children, 51% male  Adolescent, 44% male | Principal component analysis | LTPA including:  Walking  Recreational games or sport  Organised sport | Sedentary activities including:  TV  Computer  Video games | KIDMED score; Energy density; Breakfast consumption; Eating frequency; Family meal consumption; Eating home-delivered food | BMI | 1. High eating frequency, breakfast consumption and high KIDMED score  2.Low energy density, high KIDMED score  3. Sedentary activities  4. Eating family meals  5. Home food deliveries  6. Physical activities  7. Eating while engaging in other activities | 1. Average activity, average diet  2. Average activity, healthy diet  3. Sedentary, unhealthy diet  4. Inactive, unhealthy diet  5. Inactive, average diet  6. Active, average diet  7. Inactive, average diet | 46.7% |
| (38)Landsberg  2010  Germany^a^ | Median age=14.7(14.3-15.1)  n=1894  48.5% male | Two-step cluster analysis | Structured LTPA^b^  Unstructured LTPA^b^  Active travel to school^b^ | Passive travel to school  Media time including:  TV^b^  Computer^b^ | Dietary pattern; Alcohol consumption; Smoking | Age; Sex; Education; Ethnicity; Weight status^b^ | 1. Low activity and low-risk behavior  2. High media time and high-risk behavior  3. High activity and medium-risk behavior | 1. Low activity, low risk  2. Average activity, high risk  3. Healthy activity, average risk | 70% |
| (39)Laxer  2017  Canada^a^ | 9-12^th^ grade  n=18,587  51.1% male | Latent class analysis | MVPA^b^  Organized school PA^b^  Competitive school sport teams^b^  Muscle strengthening exercise^b^ | TV^b^  Video/computer games^b^  Surfing the internet^b^ | Low F&V; Low breakfast; High fast food; High corner store; High SSB; Smoker; Marijuana user; Binge drinker | BMI^b^ | 1. Traditional school athletes  2. Inactive screenagers  3. Health conscious  4. Moderately active substance users | 1. Healthy activity, average risk  2. Sedentary, low risk  3. Healthy activity, low risk  4.Average activity, high risk | 73.3% |
| (40)Laxer  2018  Canada^a^ | Mean age=14.7±0.7  n=5084  48% male | Latent class analysis | MVPA^b^  Organized school PA^b^  Competitive school sport teams^b^  Muscle strengthening exercise^b^ | TV^b^  Video/computer games^b^  Surfing the internet^b^ | Low F&V; Low breakfast; High fast food; High corner store; High SSB; Smoker; Marijuana user; Binge drinker | Sex; Race; School grade; Age; Spending money; BMI^b^ | 1. Typical high school athlete 2. Inactive high screen-users 3. Health conscious 4. Moderately active substance users | 1. Healthy activity, average risk  2. Sedentary, low risk  3. Healthy activity, low risk  4.Average activity, high risk | 83.3% |
| (41)Lazzeri  2015  Italy | 11-15 years old  n=3291  51.7% male | Factor analysis  then  k-means cluster analysis | Leisure-time PA | Computer  TV  Video/computer games | Beer consumption; Wine consumption; Spirits/liquors consumption; Alcopops consumption; Other alcohol consumption; Smoking; Current smoking; Cigarettes in last 30 days; Somatic symptoms; Psychological symptoms; Fruit consumption; Vegetable consumption; Fighting; Bullying; Sweets consumption; Soft drink consumption | Age; Sex | 1. Violent group  2. Non-drinking smoker group  3. Smoking drinker group  4. Quasi-healthy group  5. Symptomatic group  6. Screen passion group | 1. Healthy activity, high risk  2. Healthy activity, average risk  3. Average activity, high risk  4. Low activity, low risk  5. Low activity, high risk  6. Sedentary, high risk | 46.7% |
| (54)Lee  2014  United States^a^ | Mean age=15.1±1.6  n= 3717  45.9% male | k-means cluster analysis | Housework^b^ Roller-blading/cycling^b^ Sport^b^ Exercise/jogging/walking^b^ | TV^b^ Video^b^ Computer games^b^ |  | Age; Sex; Parent education; BMI^b^ | 1. LPAHSB 2. LPALSB 3. HPALSB | 1. Low/High  2. High/Low  3. Low/Low | 86.7% |
| (55)Liu  2010  United States^a^ | Mean age = 15.6  n= 13,339  49.2% male | Latent class analysis | Hobbies Sport Exercise Skating/biking Housework | TV/video |  | Age; Ethnicity; Nativity; Parental education; Household income; School status | 1. Low PA/Low SED 2. Mod PA/High SED 3. Mod PA/Low SED 4. High PA/Low SED 5. High PA(except skating/biking)/Low SED | 1. Low PA/Low SED  2. Mod PA/High SED  3. Mod PA/Low SED  4. High PA/Low SED  5. High PA/Low SED | 76.7% |
| (25)Maia  2018  Brazil | 9^th^ grade  n=109,104  47.8% male | k-means cluster analysis | PA at school  PA during leisure time | Time sitting | Healthy food intake, Unhealthy food intake, Eating behavior | Parental education; Age; Ethnicity; School status; Urbanicity | 1. Cluster 1  2. Cluster 2 | 1. Average activity, average diet  2. Sedentary, unhealthy diet | 70% |
| (42)Mandic  2017  New Zealand | Mean age=15.3±1.4  n=1300  49% male | Two-step cluster analysis | Meet PA guidelines^b^ | Meet screen time guidelines^b^ | Meet F&V guidelines^b^, Weight status^b^ | Age; Sex; Ethnicity; Neighborhood deprivation; Waist circumference; Perceived health | 1. Non-adherent, healthy weight 2. Non-adherent, unhealthy weight 3. Semi-adherent, unhealthy weight 4. Physically active, healthy weight 5. Low screen time, healthy weight 6. Healthy F&V intake, healthy weight | 1. Sedentary, unhealthy diet 2. Sedentary, unhealthy diet 3. Average activity, healthy diet 4. Active, average diet 5. Inactive, average diet 6. Sedentary, healthy diet | 63.3% |
| (53)Marshall  2002  United States | US sample  Mean age=12.9±0.9  n= 1750 41% male UK sample  Mean age=13.0±0.9  n= 744 15% male | Two-step cluster analysis | PA^b^ | TV^b^ Computer/internet^b^ Video games^b^ Homework^b^ Reading for leisure^b^ Sitting and talking/listening to music^b^ Talking on telephone^b^ |  | US sample only: Ethnicity; Age; BMI | Males 1. Techno-actives 2. Non-socializing actives 3. Uninvolved inactives Females 1. Sociable actives 2. Non-socializing actives 3. Uninvolved inactives | Males  1. High/High  2. High/Mod  3. Low/Low  Females  1. High/High  2. High/Mod  3. Low/Low | 60% |
| (43)Mikkone  2016  Finland^a^ | 16 years old  n=1625  43.8% male | Latent class analysis | MVPA | Sedentary activity including:  TV  Reading books/magazines  Computer  'Other' sedentary activities | Externalizing problems; Internalizing problems; Sleeping; Smoking; BMI |  | Boys  1. Externalizing behavior  2. Sedentary  3. Obese  4. Reference (high PA, sleep/ low internalizing, SB, BMI, smoking)  Girls  1.Externalizing behavior  2. Multiple risk behaviors  3.Obese  4. Reference (high sleep, PA/ low smoking, BMI, internalizing) | Boys  1. Average activity, high risk  2. Sedentary, Low risk  3. Average activity, low risk  4. Healthy activity, low risk  Girls  1. Healthy activity, high risk  2. Sedentary, high risk  3. Low activity, average risk  4. Healthy activity, low risk | 66.7% |
| (44)Moreira  2018  Europe | HELENA sample  12.5-17.7 years  n=2057  46.2% male  ELANA sample  13.5-19 years  n=968  46.8% male | Two-step cluster analysis | MVPA^b^ | TV^b^ | F&V; SSB | Age; BMI^b^; School status (ELANA); Family affluence (HELENA) | HELENA  Boys   1. Cluster 1 2. Cluster 2 3. Cluster 3 4. Cluster 4 5. Cluster 5   Girls   1. Cluster 1 2. Cluster 2 3. Cluster 3 4. Cluster 4 5. Cluster 5   Boys   1. Cluster 1 2. Cluster 2 3. Cluster 3 4. Cluster 4 5. Cluster 5   Girls   1. Cluster 1 2. Cluster 2 3. Cluster 3 4. Cluster 4 5. Cluster 5 | HELENA  Boys   1. High screen, average diet 2. Inactive, unhealthy diet 3. Active, average diet 4. Average activity, healthy diet 5. Inactive, unhealthy diet   Girls   1. High screen, average diet 2. Inactive, unhealthy 3. Active, average diet 4. Inactive, healthy diet 5. Sedentary, average diet   Boys   1. High screen, average diet 2. Average activity, average diet 3. Active, average diet 4. Active, healthy diet 5. Inactive, average diet   Girls   1. High screen, average diet 2. Sedentary, unhealthy diet 3. Active, average diet 4. Inactive, healthy diet 5. Sedentary, average diet | 66.7% |
| (45)Nuutinen  2017  Finland | Mean age=14.7±1.0  n=3865  47% male | k-means cluster analysis | PA | TV viewing  Computer use  Video games | Sleep duration; Discrepancy of sleep duration; Sleep quality; Junk food; F&V | Age; Educational aspiration; BMI | Boys   1. Healthy lifestyle 2. Low/moderate screen time, unhealthy lifestyle 3. High screen time, unhealthy lifestyle   Girls   1. Healthy lifestyle 2. Poor sleep, unhealthy lifestyle 3. High screen time, unhealthy lifestyle | Boys   1. Active, healthy diet 2. Inactive, unhealthy diet 3. High screen, unhealthy diet   Boys   1. Active, healthy diet 2. Inactive, unhealthy diet 3. High screen, average diet | 63.3% |
| (46)Nuviala  2009  Spain | 10-16 years  n= 1829  48.3% male | Two-step cluster analysis | LTPA | TV  Computer Schoolwork |  | Sex; Education level | 1. Passive leisure time 2. Active leisure time | 1. Low/High  2. High/Low | 40% |
| (47)Ottevaere  2011  Europe | 12-18 years  n= 2084  45.6% male | Two-step cluster analysis | MVPA^b^ | Sedentarism (inc. TV, computer games, video games, internet for leisure, internet for study, homework) ^b^ | DQI-A | Age; Sex; BMI; Parental education^b^; Family affluence^b^ | 1. Unhealthy 2. Sedentary 3. Active, low diet quality 4. Inactive, high diet quality 5. Healthy | 1. Inactive, unhealthy diet  2. Sedentary, average diet  3. Active, unhealthy diet  4. Inactive, healthy diet  5. Active, average diet | 80% |
| (12)Patnode  2011  United States | Mean age=14.7±1.8  n= 720  48.9% male | Latent class analysis | MVPA^b^ Traditional sport^b^ Fitness activities^b^ Other sports/physical activities^b^ Chores/work^b^ | TV  DVD/video  Video/computer games Internet/computer Talking/texting on phone Reading/homework |  | Sex; School grade; Parental education; Ethnicity; Household composition; Free/reduced lunch; Weight status^b^ | 1. Active 2. Sedentary 3. Low media/functional activity | 1. High/Low  2. Low/Low  3. Mod/Low | 60% |
| (56)Perez-Rodrigo  2011  United States | Mean age=12.7±2.7  n=415  62.2% male | Two-step cluster analysis | MVPA^b^  Active travel^b^ | TV  Computer games  Video games  Internet for leisure  School related internet  Study/homework | Diet patterns; Sleeping habits | Age; Sex; Parental education; Family SES; BMI | 1. Unhealthier lifestyle pattern  2. Healthier lifestyle pattern | 1. Inactive, unhealthy diet  2. Active, healthy diet | 63.3% |
| (48)Spengler  2012  Germany | Mean age=13.7±1.9  n=1643  50.6% male | Two-step cluster analysis | Sport club^b^ LTPA^b^ | TV  Video games Computer | HuSKY | Sex; Age; SES; Migration background; Weight status^b^ | 1. High PA/Mod media use and HuSKY 2. High HuSKY/Low PA and media use 3. Low PA/Low HuSKY/High Media use 4. Low PA/HuSKY/Media use | 1. Average activity, average diet  2. Inactive, healthy diet  3. High screen, unhealthy diet  3. Inactive, unhealthy diet | 70% |
| (49)Spengler  2015  Germany | Mean age=13.8±1.9  n=2083  49.5% male | Two-step cluster analysis | Sport club^b^ LTPA^b^ School PA^b^ | TV viewing Video games Computer |  | Sex; Age; SES | Males 1. Low PA/Low media 2. Low PA/Med TV & very high games 3. Low PA/High TV & computer 4. Low PA/Very high TV & games & computer 5. Very high leisure PA/Med media 6. Very high school PA & Med sports club PA/Med media 7. Very high sport club PA/Med media 8. High sport club PA/Low media Females 1. Low PA/Low media 2. Low PA/High TV 3. Low PA/Med TV & Very high computer 4. Low PA/Med TV & High games & Med computer 5. Very high leisure PA/Low media 6. Very high school PA & Med sport club PA/Low media 7. Very high sport club PA/Low media | Males  1. Low/Low  2. Low/Mod  3. Low/High  4. Low/High  5. High/Mod  6. High/Mod  7. High/Mod  8. High/Low  Females  1. Low/Low  2. Low/High  3. Low/High  4. Low/High  5. High/Low  6. High/Low  7. High/Low | 70% |
| (50)Valencia-Peris  2016  Spain | 12-18 years old  n=1516  100% female | Self-organizing maps (SOM) analysis | MPA  VPA  MVPA | TV  Computer for playing  Computer for leisure  Computer for homework  Overall computer use  Video games  Overall sedentary screen media |  |  | 1. Temperate-media active  2. Prudent-media active  3. Compulsive-media active  4. Not-named  5. Not-named  6. Not-named  7. Not-named  8. Not-named  9. Not-named | 1. High/Mod  2. Low/Low  3. Low/High  4-9 unknown | 60% |
| (51)Veloso  2012  Portugal | Mean age=14.8±1.1  n=3069  45.9% male | Cluster k-means non-hierarchical method | MVPA Intense leisure time exercise | TV  Video/computer games Computer for internet/homework | Dieting | Sex; School grade; Weight status; Self-regulation; Motivation; Body satisfaction; Communication with parents; Relation with family/classmates; Liking school | 1. Active gamers 2. Healthy 3. Sedentary | 1. High screen, unhealthy diet  2. Active, healthy diet  3. Inactive, average diet | 46.7% |
| (52)Wang  2012  Singapore | 10-16 years  n=847  35% male | Latent profile analysis | PA (included exercise, sport, dance, general PA)^b^ | Computer/internet^b^ Video games^b^ Homework^b^ Reading^b^ Sitting talking^b^ Telephone^b^ TV^b^ |  | Sex; School grade | 1. High homework, low video game, low TV  2. Low PA and SB  3. High computer, high video game and high PA  4. Low video game, high TV, low PA 5. High video game and high PA | 1. Mod/Mod  2. Low/Low  3.High/High  4. High/Low  5. High/High | 60% |

^a^ indicates longitudinal study design, all others are cross sectional

^b^ indicates validity/reliability of instrument used

PA: Physical Activity, MVPA: Moderate-to Vigorous-Physical Activity, MPA: Moderate-Physical Activity, VPA: Vigorous-Physical Activity, LTPA: Leisure-Time Physical Activity, PE: Physical Education, TV: Television, BMI: Body Mass Index, SSB: Sugar Sweetened Beverages, F&V: Fruit and Vegetable intake
